# Practical Observations on the Treatment of the Diseases of the Prostate Gland. Illustrated by Copper-Plates

**Published:** 1813-03

**Authors:** 


### CRITICAL ANALYSIS

### *OF RECENT PUBLICATIONS*

IN THE

### DIFFERENT BRANCHES OF PHYSIC, SURGERY, AND MEDICAL PHILOSOPHY.

---

*Practical Observations on the Treatment of the Diseases of the Prostate Gland. Illustrated by Copper-plates. By* EVERARD HOME, Esq. F.R.S. Serjeant-Surgeon to the King, and Surgeon to St. George's Hospital. 8vo. Lond. 1811. pp. 272–lxxx. 10 Plates. Nicol.

*A Review of Mr. Everard Home's Practical Observations on the Diseases of the Prostate Gland, and of his important Anatomical Discovery. By* JESSE FOOT, Surgeon. 8vo. Lond. 1812. pp. 39. Highley.

FEW works of modern surgery have excited more attention, enjoyed more fame, or suffered more reproach, than these "Practical Observations," by Sir Everard Home. Three causes, principally, have co-operated to produce this effect: the celebrity of the author—the importance of the subject, rendered more interesting by an asserted discovery—and the animadversions of Mr. Foot. Had we been disposed to animadvert on this production of a popular surgeon, we should have been completely anticipated by Mr. Foot, the lacerating severity of whose strictures have left nothing to be added by the most caustic critic. As it has, however, always been our desire to bring into public notice useful facts and practical observations, rather than wield the castigating pen of criticism, right glad are we to escape from the task; and, while we confine ourselves to an analysis of this, with all its blemishes upon it, useful volume, we delegate that thankless office to our brother Reviewer, Mr. Jesse Foot, who is as keen a demolisher

"As slashing Bentley with his desperate hook."

The

The volume of Sir Everard Home is divided into ten chapters, some of which have a subdivision of sections. The first Chapter treats "*On the Discovery of a middle Lobe of the Prostate Gland*," and commences with the publication of a paper read to the Royal Society, in February 1806, containing a history of the discovery of this middle lobe, a description of the lobe itself, and a plate illustrative of this description, and shewing "the external appearance of the middle lobe when the vesiculæ seminales and the vasa deferentia, under which it is situated, have been removed.". As the peculiarity of structure in the Prostate Gland, here asserted by the author, forms the basis of all his reasonings and conclusions in the subsequent part of the volume, we shall give the facts respecting this peculiarity, and the history of the discovery, in his own words.

"Although," he says, "a great part of my time has been for many years occupied in attending patients laboring under complaints of the bladder and urethra, and my opportunities of examining these parts after death have been very frequent, my attention has been always so much employed on the modes of emptying the bladder, (an operation which in many cases is attended with considerable difficulty,) that it never occurred to me to institute an inquiry for the purpose of attaining an accurate knowledge of the origin of the disease until the month of December 1805.

"At that time my attention was directed to this subject, by the following circumstances. In the examination of the prostate gland of an elderly person, who had died in consequence of this part being diseased, the nipple-like process was found very prominent, and a bridle, nearly a quarter of an inch in breadth, extended from the middle line of the tumor to the bulb of the urethra, where it insensibly disappeared. The usual rounded projection of the verumontanum was not visible: it had wasted away, and the remains were concealed in the fold forming this bridle, which at that part was not thicker than any other. The space between the tumor in the bladder, and the bulb of the urethra, was unusually short, which is the reverse of what is commonly met with in old men; so that this bridle appeared to have drawn the bulb towards the tumor, and shortened the membranous part of the canal.

"As this was an unusual appearance, it led me to consider it with attention, and to ask if other anatomists had noticed it; which, as far as my inquiries have gone, has not been the case. The bridle had evidently been formed by the membrane of the bladder adhering firmly to that part of the prostate gland composing the tumor, which it consequently followed in its future increase, and drew up after it the membrane of the urethra. In this way the fold had in time become nearly a quarter of an inch broad, and was continued of the same breadth to the bulb, where the lining of the urethra being more attached to the surrounding parts, it did not admit of being drawn up.

"This appearance of a bridle is more or less met with in all the cases, in which the nipple-formed process occurs, but in a much smaller degree, and does not extend further forwards than the verumontanum.

"To satisfy myself how this tumor was formed, it became necessary to examine the prostate gland in its natural state; and ascertain whether there is any part sufficiently detached to move independent of the rest of the gland, and so explain the appearances which had been met with in this particular case.

"My professional avocations not affording time to make the dissections requisite for this purpose, Mr. Brodie, who is engaged in teaching anatomy with Mr. Wilson, in Windmill-street, whose knowledge of the subject fitted him for the task, and whose zeal for the improvement of his profession made him willingly undertake it, gave me his assistance, and took the whole of that labor on himself.

"While dissecting the parts for this purpose, the urinary bladder was distended with water, and the surfaces of the prostate gland, vesiculæ seminales, and vasa deferentia, were fairly exposed. This being done, the vasa deferentia, and vesiculæ seminales, were carefully dissected off from the bladder, without removing any other part. These were turned down upon the body of the prostate gland. An accurate dissection was then made of the circumference of the two posterior portions of the prostate gland, and the space between them was particularly examined. In doing this a small rounded substance was discovered, so much detached that it seemed a distinct gland, and so nearly resembling Cowper's glands in size and shape, as they appeared in the same subject, in which they were unusually large, that it appeared to be a gland of that kind. It could not, however, be satisfactorily separated from the prostate gland, nor could any distinct duct be found leading into the bladder.

"A similar examination was made of this part in five different subjects. The appearance was not exactly the same in any two of them. In one there was no apparent glandular substance, but a mass of condensed cellular membrane: this, however, on being cut into, differed from the surrounding fat. In another there was a lobe blended laterally with the sides of the prostate gland. These facts are mentioned in proof of its not being always of the same size, or having exactly the same appearance; this is found also to be the case with Cowper's glands; they are sometimes large and distinct; in other subjects they are scarcely to be detected, and in others again are in all the intermediate states. The most distinct and natural appearance of this part was in a healthy subject, twenty-five years of age, of which the following is an account. On turning off the vasa deferentia, and vesiculæ seminales, exactly in the middle of the sulcus, between the two lateral portions of the prostate gland, there was a rounded prominent body, the base of which adhered to the coats of the bladder. It was imbedded not only between the vasa deferentia and the bladder, but also in some measure between the lateral portions of the prostate gland and the bladder, since they were in part spread over it, so as to prevent its circumference from being seen, and they adhered so closely, as to require dissection to remove them; nor could this be done beyond a certain extent, after which the same substance was continued from the one to the other. This proved to be a lobe of the prostate gland; its middle had a rounded form, united to the gland at the base next the bladder, but rendered a separate lobe by two fissures on its opposite surface. Its ducts passed directly through the coats of the bladder, on which it lay, and opened immediately behind the verumontanum. By means of this lobe, a circular aperture is formed in the prostate gland, which gives passage to the vasa deferentia.

"The appearance of this lobe has been since examined in a subject twenty-four years of age, and it was found still larger and more distinct. A representation of it is given in Plate I."

The portion of the prostate gland, described in the above passage, had, previous to this investigation, from the obscurity of its situation, escaped remark; and, did it not, when morbidly enlarged, sometimes completely shut up the urethra, as our author asserts, it would hardly deserve attention. If, however, it does at any time produce this important effect, it becomes an object of the deepest interest. And, if this discovery "enables us very clearly to understand the nature of a disease, which it was not possible to have a correct idea of, when we were ignorant of the existence of the part in which it takes place," it involves a still deeper interest, and a more important subject of solicitude and earnest consideration.

The second Chapter, of six sections, inquires into "*the enlargement of the middle Lobe of the Prostate Gland, and the different effects it produces.*"

The first section, "*On the different Stages of Enlargement, in which the middle Lobe either impedes the flowing of the Urine, or entirely puts a stop to it,*" contains some interesting facts, and hazards some opinions that will be controverted. It appears, from the experience of the author, that the middle lobe of the prostate is not liable to be enlarged till the latter periods of life.

"From 15 to 40 years of age, the most common disease of the urinary organs is strictures in the urethra, and after that age persons are not only less liable to that complaint, but, if no serious consequences have been previously produced by it, there is little reason to apprehend them afterwards, unless some unusual circumstances occur to aggravate the original complaint.

"In advanced life, on the other hand, the prostate gland, which in all the earlier periods was little liable to disease, is more subject to be affected than most other parts of the body; and it is a rare occurrence for a man to arrive at 80 years of age, without suffering more or less under disease of this part. From its frequent occurrence, perhaps we may be justified in believing that it is alluded to in the beautiful description of the natural decay of the body, in the Bible, in in the book of Ecclesiastes, the 12th chapter, the 6th verse, where it is written, 'or the pitcher be broken at the fountain, or the wheel broken at the cistern.' Expressive of the two principal effects of this disease, the involuntary passing of the urine, and the total stoppage."

When the middle lobe does begin to enlarge, it presses inwards towards the cavity of the bladder, and, as it increases in size, it projects into that cavity in the form of a nipple; but, as it further enlarges, it loses that shape, and becomes broader from side to side, and forms a transverse fold by pushing forward the membrane connecting it with the lateral lobes. As the enlarged middle lobe and the transverse fold are situated immediately behind the orifice of the urethra, they are pushed forward before the urine in every attempt made to void it, acting like a valve, and closing the opening, until the accumulating urine alters the form of the bladder by distention, in such a way, that some of that fluid escapes, though the bladder is not completely emptied. This morbid state of the prostate is very different from that which arises in earlier life, as the consequence of stricture; the enlargement of the lateral lobes is irregular, and the projection of the middle lobe into the bladder, with the transverse membranous fold, is sufficient to produce a complete retention of urine.

Section 2 treats "*on the Effects produced on the Secretion of the Gland*," by the enlargement of the middle lobe. As the body of the gland and the lateral lobes are enlarged as well as the middle lobe, it is not understood that the diseased state of the latter peculiarly contributes to the abundant viscid secretion which takes place. This secretion occasionally almost equals the quantity of urine voided, and is extremely tenacious and ropy.

Section 3, "*on the Effects produced on the Coats of the Bladder*," shews that inflammation, first of the internal membrane of the bladder, in immediate contact with the tumor, extending afterward over its whole surface, and extreme irritability of that organ, are the consequence of the enlarged middle lobe. The property in the urinary bladder of admitting of extraordinary dilatation without much injury to the constitution, or great distress to the patient, is a fact that deserves attention. Under these circumstances the tumor of the bladder has been mistaken for hydrops. It is here related, that Mr. Hunter, in one instance of this state, was employed to tap the patient. "He performed the operation, but from the smell of the fluid found at once the mistake; and, with a presence of mind that did him credit, put put a plug into the canula, made an incision into the cavity of the abdomen, and secured the wound in the bladder."

Section 4 is "*on the Disposition which this Disease gives for the Formation of Stone.*" This diseased state of the prostate sometimes becomes an indirect cause of the formation of a urinary calculus, by preventing a complete emptying of the bladder. In the dregs of the urine thus retained, a calculus is formed on a nucleus of the ammoniaco-magnesian phosphate and mucus. It is worthy of remark, "that the enlargement of the middle lobe of the prostate gland not unfrequently produces a cure of the symptoms of the stone, by preventing the calculus coming in contact with the neck of the bladder."

The 5th and 6th Sections describe the effects of this disease upon the secretion of urine, and the suppression of that secretion, as one of the causes of death, when the retention in the bladder has been of long continuance. Patients who labor under this disease, and do not completely empty the bladder, secrete less urine than usual; and, when the bladder is relieved by art, the secretion returns to its natural standard. In suppressions of urine, where no local injury of the bladder, &c. appear of sufficient magnitude to occasion death, and after the local symptoms have subsided, the constitution becomes disturbed: there is quick pulse; great thirst; general irritation; anxiety of mind; the tongue is covered with a brown fur; and the patient becomes delirious, with intervals of stupor. Under these symptoms the patient dies, and is not uncommonly believed to have died of typhus. For the explanation of these phenomena, and this fatal consequence of suppression of the secretion of urine, we must refer to the author's observations, and the accompanying cases.

In the third Chapter, consisting of four Sections, the mode of treating cases of enlargement of the middle lobe of the prostate gland is discussed.

The means to be employed in the earliest stage of this disease, are treated of in the first section of this chapter. When the membrane of the bladder is only pushed forward by the lobe beginning to enlarge, and no absolute obstruction to the passing of the urine is brought on, bleeding from the loins, opiate glysters, Dover's powder internally, the tepid hip-bath for 15 minutes, once in 24 hours, at 94° or 95°, quietness and abstinence, are the means to be employed. "On no account, in this stage of the disease, should catheters or bougies be introduced, especially those of the metallic kind, since when done in the most skilful manner they must produce a degree of disturbance which the parts are not in a state state to bear; and, if an instrument is unskilfully passed, it will increase the swelling, and bring on a complete retention of urine."

Sir Everard Home, in the former part of his work, has observed, that strictures in the urethra seldom appear *first* after 40 years of age. In this section he states this position still more strongly, "Attacks of this kind," (frequent irritation to pass urine, which is voided with difficulty, the consequence and symptom of the first stage of the morbid enlargement of the middle lobe,) "are generally attributed by the patients themselves to a stricture in the urethra, and therefore they propose having a bougie passed; but, if it is found on inquiry, that in no former part of his life the patient has had such a disease," (stricture,) "and he is now advanced in years, *it may be laid down as a certainty, that it has not now taken place.*"

Section 2 of this Chapter is of great interest, as coming from an artist of such celebrity; it is "*on the Mode of drawing off the Urine.*" In order to do justice to this, we shall quote as much of the author's own statement as our limits permit.

"In the introduction of the catheter there are three things to be attended to: the first is, to avoid bringing on a spasm of the urethra; the second is, conducting the point of the instrument over the prominence at the neck of the bladder; and the third, to employ an instrument that is fitted to be retained in the bladder, should much difficulty have occurred in the introduction, as less disturbance is found to arise from an instrument remaining in the bladder, than is produced by repeating the operation of introducing it, where any degree of violence is committed upon the parts.

"The instrument should be very soft and smooth, to prevent its disturbing the urethra, rounded at the point, and as large as the canal will easily admit, that it may more readily disengage itself at the turn into the bladder: the apertures in its sides should be wide, to prevent their being clogged by mucus or blood; and it should be pliant, that it may adapt itself to the form of the parts, and give little disturbance while retained in it. Besides these properties, there is another, which it is very desirable that it should possess; this is, a permanent curvature at the point, even to a greater degree than is usually given to the common silver catheter.

"The only instrument with which I am acquainted, capable of possessing all these requisites, is the elastic gum catheter; but it requires years before it can be made to acquire the permanent curvature which I have described, and for that purpose it must be constantly kept upon an iron stilet of a proper shape. This has not been attended to; and the makers neither in England nor France give their flexible catheters a proper shape, but either keep them quite straight, or so curved, that the curvature is not regularly continued to the point, and is therefore of no use in practice.

"This

"This has arisen from surgeons being in the habit of only using these catheters with stilets; not aware of the advantage to be derived from using them in this particular disease in a more pliant form, and, therefore, not having given directions either to the makers or venders upon the subject.

"This has been a great oversight, and has deprived the flexible gum catheter of one of its greatest excellencies, both in the present disease, and in cases of stone attended with great irritation; since, in many instances of both complaints, an instrument so prepared will admit of being introduced without pain into the bladder, when a common catheter cannot be passed at all, or with much pain to the patient, and no small difficulty to the surgeon.

"For the last ten years, I have had a large supply of catheters carefully formed into a proper curve, and with them have been able to draw off the water in cases in which other surgeons, who did not possess them, have necessarily failed."

The flexible gum catheter is the instrument preferred even in sounding for the stone; and the author mentions some instances of his success with this instrument, when all previous attempts with others had failed. In the particular disease which is the subject of this volume, an enlargement of the middle lobe of the prostate, he considers the elastic gum catheter to be the only safe and manageable instrument. With respect to the use of this instrument, the following practical observation is made: "In some cases, a flexible gum catheter with a stilet, cannot pass along the urethra on account of spasm; and, although without a stilet, it readily goes on to the neck of the bladder, unless it has a permanent curvature that will keep its form when opposed by a certain degree of resistance, it cannot be conducted into the cavity of the bladder. A case is given to illustrate this fact. The following observations on the manual dexterity required for the introduction of instruments into the bladder by the urethra, and upon some other points in this delicate operation, may be gratifying to our readers.

"In passing the catheter, a degree of nicety and lightness of hand is necessary, which can be no where acquired but in the school of experience, and that in cases of this particular disease. It is not, therefore, to be expected that many should arrive at it, since the opportunities afforded must so frequently be insufficient for the purpose. An eminent engraver, on whom I passed a catheter, after unsuccessful attempts had been made by others, told me that he saw by the light mode of handling the instrument that I was master of it; and that, in his own art, the graver required a management in the hand which it was difficult to learn, and only to be acquired by practice. The catheter should be introduced either towards the right or left side, with the handle nearly in a horizontal line; and, when it reaches the membranous part of the urethra, the handle should be gently and gradually brought towards the perpendicular line, the point all the time time being kept in motion; and when it is nearly upright the handle should be depressed: where the flexible catheter has no stilet, a good deal of dexterity is often required. The great advantage of passing the instrument in a lateral direction, is, that the point is by that means guided into the space between the lateral and middle lobe of the prostate, where there is a groove, along which it may be directed, between these two projecting parts, into the cavity of the bladder. In this part of the operation, the greatest nicety is required, for, if the point is too much pressed on, it is entangled in the membranous fold between the two lobes; and this very frequently happens: when this takes place, a second and third attempt made in the same way will most commonly carry the point into the same spot. There will, therefore, be an advantage in partly withdrawing the instrument, and trying to introduce it on the opposite side, where the same thing may not occur.

"If the catheter without the stilet cannot in this way be made to pass, it must be tried with a stilet; and, if it is still prevented from going further than before, a finger introduced into the rectum and pressing upon the curved part of the catheter may give it a right direction so as to guide it into the bladder.

"Wherever any degree of difficulty has occurred in passing the instrument the first time, it is always prudent to retain it in the bladder, for, unless it is introduced again in five or six hours, its having been introduced at all is of little use; for in that period the bladder is generally as full as it was when the water was drawn off. Whether this arises from the accumulated fluid in the kidneys passing into the bladder, or from the pressure taken off from the mammæ, making them secrete more freely, is immaterial; but the fact is of importance, since it makes it necessary again to empty the bladder, and the disturbance the parts received in the first operation, cannot so soon have gone off, and therefore must increase the difficulty in the second attempt.

"Much has been said respecting the posture of the patient most favorable for the instrument being passed. It appears to me, that, in this respect, the ease of the patient is to be principally considered; for, in that position which is most easy, the parts will be in the most tranquil state. The standing position I prefer, but not to that degree to make a patient get up when lying in bed. Where it is necessary to introduce the finger per anum, of course the recumbent posture must be employed.

"When the instrument is passed, and it is determined to keep it in the bladder, it is necessary to observe when the cavity is empty, how much of the catheter projects beyond the external orifice at the glans penis, and cut off all that is superfluous, only leaving an inch, by means of which it may be secured on each side to prevent it from either coming out or going further into the bladder; the orifice is to be stopped with a wooden peg, as corks are apt to break: and this is to be withdrawn whenever the inclination to make water comes on.

"The best mode of securing the catheter, is to apply, in the usual way, a common T bandage, the longitudinal band of which is divided up the middle, into two portions, one of which lies in each angle between between the scrotum and thigh, and furnishes a fixed point, to which the catheter may be secured by ligatures. The catheter, thus secured, is less liable to slip out of its place than if it was merely fastened by a ligature round the penis.

"The time the instrument is to remain in the bladder must depend upon circumstances. If it can be kept there without great inconvenience, it should be left in three or four days; but, if the patient is much troubled with erections, or the bladder is rendered very irritable, it had better be taken out; and, in general, it will be found that there is not the same difficulty in passing it the second time as the first. Whether it is afterwards to be kept in, or to be introduced each time that the urine requires to be drawn off, must be determined by the degree of facility with which the catheter can be passed."

Section 3, "*On the circumstances under which the Swelling of the Prostate Gland subsides, and the Bladder recovers its tone,*" shews that these circumstances are, principally, a removal of the irritation occasioned by the retained urine. To effect this, it is necessary that the urine be drawn off every eight, six, or four, hours, as the secretion is more or less rapid.

The 4th Section consists of Cases to illustrate this mode of treatment.

The fourth Chapter is "*On the enlargement of the middle Lobe of the Prostate Gland, when it comes on while the patient is laboring under Stricture in the Urethra.*" The three sections of this chapter explain the nature of the complication of the two diseases, the mode of treating them when so complicated, and a series of cases in which the treatment of this complication of the two diseases is illustrated.

Ten cases of different degrees of enlargement of the Prostate Gland, in which the Parts were examined after death, illustrated by plates, make the fifth Chapter.

The sixth Chapter details the effects of different kinds of Injections into the Bladder upon the symptoms of this Disease. The injections used were olive oil, camphorated oil, laudanum, linseed oil, tepid water, but with a success that did not answer the expectations formed.

The enlargement of one of the lateral Lobes of the Prostate Gland, producing painful sensations in the rectum, is the subject of the seventh Chapter.

An enlargement of one of the lateral lobes of the Prostate, in such a direction as to press backwards upon the rectum, without affecting the orifice of the bladder, and unaccompanied by any swelling of the middle lobe, is a disease of such rare occurrence, that, in the extensive practice of our author, only two cases have fallen under his notice.

The tumid lobe projecting into the rectum, which lies immediately in contact with it, gives this disease a close affinity in effect to the swelling of the middle lobe into the bladder, producing an obstruction to the passing of the fœces, similar to the difficulty which the bladder experiences in emptying itself of urine, when the canal is nearly closed by the morbid tumor in the latter instance. The following particulars respecting a disease, but little known certainly, perhaps not before distinctly pointed out, are too valuable to be omitted.

"When the parts are not disturbed, no symptoms take place; but the act of going to stool, by pressing the membrane which lines the intestine between the enlarged lobe and the contents of the bowel, brings on uneasiness. The same effect is produced by walking, exercise, and any other bodily exertion: this effect of fatigue does not, however, immediately follow it, but comes on several hours afterwards, so that it is not uncommonly experienced in the following night. That the symptoms arise from the pressure on this part, is clearly ascertained, from similar uneasiness being brought on by pressing the finger upon this particular spot; and even then it is entirely referred to the intestine, not to the gland itself. In all these respects, this disease corresponds with what takes place in the enlargement of the middle lobe; nor is there any reason to believe, that, in either case, the symptoms are much aggravated by an increased sensibility of the internal structure of the gland itself.

"The symptoms are, great distress in the act of going to stool, followed by the most painful kind of tenesmus, which is increased in violence by the efforts it almost compels the patient to make in straining, still further to empty the intestine. When these exertions are no longer made, an aching uneasy feeling continues in the parts for some hours. These symptoms very much resemble those in the bladder brought on by the enlargement of the middle lobe, in the act of making water, and after a small quantity is voided. In one case, they misled the surgeon into a belief that there must be a stricture in the rectum, and the use of large bougies was recommended, and actually adopted; nor did the facility of passing them make the practitioner see his error; but the pain they occasioned made it impossible for the patient to persevere in their use.

"In this disease, as in the other, the principal treatment I have to recommend is, to relieve the parts from pressure by every possible means; and it is very satisfactory to be able to state, that, by a perseverance in such treatment, both the patients in which this disease has occurred have got well: the symptoms not only yielded, but the swelling which projected into the rectum subsided."

The applications which appeared to be of most benefit were suppositories of extract of hemlock, either with or without opium, enema of warm water, both as a tepid bath to the bowels, and a means of procuring regular evacuations without disturbance to the part affected. The use of the tepid hip-bath with salt and water, once in twenty-four hours, at 92°, was considered to be serviceable, at least grateful to the patient's feelings. The following case will exemplify the method of treatment.

"*Case.*—A. B., thirty-six years of age, in the year 1803, first felt a violent throbbing pain in the rectum: this came on in irregular paroxysms, and occurred more commonly in the night. After going to stool, there was a sense of tenesmus, of the most distressing kind: it was brought on by the necessary effort that he made in emptying the bowels, which he had occasion to do every morning, and lasted the greater part of the forenoon. The sensation was so uneasy as to render him unable to attend to business. Several internal medicines were employed, without any beneficial effect. Glysters of lime-water were retained all night in the quantity of two ounces, but were of no use. Sedative linaments, and fomentations, were applied to the perinæum, but gave no relief. Bougies were passed up the rectum, but aggravated all the symptoms.

"After the complaint had continued for a year, I was first consulted, and, on examining the rectum, found a prominence on the prostate gland: by pressing on this, I brought on the symptoms to which the patient was liable from the disease: there could, therefore, be no doubt that it was the seat of the complaint. I recommended the use of suppositories composed of extract of opium, and extract of hemlock. The quantity of opium was two grains to four or five hemlock. I directed a clyster of a pint of warm water to be injected every morning to procure a stool, and the tepid salt-water hip-bath to be used every forenoon for ten minutes, at 92°. In the course of fourteen days, the tumor was evidently diminished in size, and the uneasy sensations considerably relieved, but, as the opium was found to affect the head, and made the patient very uncomfortable, its use was omitted. He persevered in the use of the extract of hemlock alone, and increased the temperature of the hip-bath to 94° for fifteen minutes. In the course of six months his symptoms were entirely removed, and, when the parts were examined by the finger, little or no swelling could be felt.

"This gentleman has had no return of his symptoms since the year 1805, till 1810, when they were felt in a slight degree, but the attack was so mild as not to induce him to take up any mode of treatment."

The eighth Chapter is "*on the Inflammation of the Verumontanum.*"

The symptoms of this disease are sometimes of the most painful kind, suddenly changing to the most tormenting itching and worrying sensations. The description which individual patients give of these symptoms would fill a volume, and they vary according to the peculiarities of each individual constitution. They are always increased by sitting and standing, and relieved by an horizontal posture, although not removed.

Abscess and Ulcer of the Prostate Gland are treated of in the ninth and tenth Chapters.

There is nothing in these chapters which will warrant us

 further further to extend our analysis: we shall here, therefore, close it with a case of ulcer in the prostate, on account of the singularity of its cause.

"A gentleman, fifty-nine years of age, who had passed many irregularly-formed portions of gravel, and some of them with great difficulty, their form retaining them several days in the urethra, was attacked with a discharge of glary mucus, attended with frequency in making water, which in no way could be accounted for: the quantity of mucus increased, and it became more tenacious; nothing that was administered checked it in the smallest degree; it went on for a year and a half, and at the end of that time he died of another disease. Upon examining the parts after death, two portions of gravel of very irregular forms were found imbedded in the prostate gland, hardly perceptible in the urethra, only one small point projecting beyond the surface of the membrane: when these were removed, there was a cavity nearly the depth of the thickness of the gland, containing an imperfectly-formed matter, with which the calculi were surrounded.

"In this case, it was impossible during life to ascertain the cause of the symptom, nor could any means of relief have been administered, had it been known.

A minute and useful description of the ten plates, and tables shewing the irregularity of the secretion of urine, in patients who labored under a disease of the prostate gland, occupy the remainder of the volume.

---

Whoever places himself on a pedestal and courts public investigation, must submit to have his proportions, his contours, and his general symmetry, examined by the eagle eye of criticism. Moderation and candor, he must expect, will hide their diminished heads before severity and perhaps prejudice. Sir Everard Home is placed high on the pedestal of public opinion—Mr. Foot will supply the rest. His originality, his opinions, his practice, and his principles, all are examined with a bold and free spirit. With deep and painful incisions, this reviewer, when he intends to remove or correct the rotten, often endangers the sound, parts. His scalpel knows no bounds; when it cuts like a saw, he applies it with a heavy hand. Not, however, that it always lacerates, for at times it dissects with the razor keenness of wit. But we have promised that he shall speak for himself, and from that promise we must not depart.

On the discovery of the "*middle Lobe of the Prostate*," after some, not very civil, glances, *en passant*, at the worthy President of the Royal Society, the Reviewer says,

"Six years have elapsed, and not one single syllable has been said, nor one observation published, in behalf of this new anatomical discovery, so that the author has been driven to the mortifying necessity of being the herald of his own fame thus at second-hand."

On

On some of the appearances, qualities, and effects, of the middle lobe, Mr. Foot makes the following remarks:

"This newly-discovered lobe or gland, exhibits itself, according to Mr. Everard Home's plate, and his description, in the middle of the upper circumference of the prostate gland. The appearance is there seen to be angular, the apex of the angle pointing towards the verumontanum, and the base rounding in a line with the upper circumference of the prostate. As there cannot be a doubt but that as much of it is presented, as has been his design to be demonstrated, so by virtue of this plate we may infer that its proportion to the prostate might be more than a fortieth part, perhaps a fiftieth, as this diminutive stranger is there to be seen.

"This diminutive stranger is concealed from Mr. Everard Home's sight anteriorly by the insertion of the vesiculæ seminales into the prostate, but it accommodates the rout of the vesiculæ seminales by graciously permitting them to travel round it. It is, as he says, connected firmly with the prostate anteriorly, but it is distinct posteriorly: neither Morgagni, nor Hunter, nor any other anatomist past or present, had any idea of it.

"This diminutive stranger possesses, perhaps, as many powerful and mischievous qualities as ever fell to the lot of any component part of the human frame. Whenever it be irritated or diseased, it throws out a process in the form of a nipple at first, independent of the prostate, which, solely from an increase in its dimensions, produces the most fatal cases hitherto known of diseases of these parts, and without which such cases never could have occurred."

The uncertainty into which Sir Everard Home had fallen with respect to the actual quality and name of the part he claims to have discovered, is seized upon with avidity.

"The discovery of this diminutive stranger was made in consequence of an examination of the prostate gland in its natural state.

"'An accurate dissection was made of the two posterior portions of the prostate gland, and the space between them was particularly examined. In doing this, a small rounded substance was discovered, so much detached that it seemed a distinct gland, and so nearly resembling Cowper's glands in size and shape, as they appeared in the same subject, in which they were unusually large, that it appeared to be a gland of that kind.'

"Here is described a distinct well-defined gland, large enough to be seen then and hereafter by every attentive eye, so large as Cowper's glands at their largest size: here is the gland never discovered before! It is here not even said to be a lobe of the prostate, but it is a gland, a perfect gland, detached to all appearance, distinct in every point!

"As if this had been too much, then comes another discovery—

"'It could not (the diminutive stranger), however, be satisfactorily separated from the prostate gland, nor could any distinct ducts be found leading to the bladder.'

"In order to save appearances, and to provide himself with an apology apology to any future anatomical inspector and inquisitor that had gone especially in search after this newly-discovered glandular substance, similar to and distinct as Cowper's glands, or this middle lobe so annexed to the prostate gland, that dissection cannot separate it, as he has also described it. Mr. Everard Home proceeds to state subsequent examinations.

"'A similar examination was made of this part in five different subjects. The appearance was not exactly the same in any two of them: in one there was no apparent glandular substance, but a mass of condensed cellular membrane; this, however, on being cut into, differed from the surrounding fat. In another there was a lobe [not gland] blended laterally with the sides of the prostate gland.'

"One could readily anticipate, from the word [however], that the middle lobe, or the gland, would not be found in this research—It was not to be.

"At length, after having thus searched, and thus defined, this gland, when it could be found, to be similar to Cowper's glands; and after having also defined it to be a lobe, Mr. Everard Home made it his adoption by calling it hereafter a lobe, the middle lobe of the prostate gland; and that the prostate gland, to which it is so connected that dissection cannot separate it, should have an allotment in this discovery, he has set his baptismal nomenclature to work. The right and left portions of the prostate gland are to be called the right and left lobes, and this diminutive stranger, the middle lobe; that is, when it can be found."

It is very unusual with the author of the "Practical Observations" to indulge in any thing like the flowery eloquence so often found in the pages of juvenile writers: in one instance, however, he has fallen into this error, where he conjectures that the "beautiful description of the natural decay of the body," in the book of Ecclesiastes, applies to this morbid state of the prostate gland. The Reviewer does not fail to notice this, and accuses him of poaching in the pages of Dr. Mead.

"If Mr. Everard Home means, by this partial piece of stealth, to establish the predominant occurrence of these complaints in old age over all others enumerated out of Ecclesiastes by Mead, he has been attempting to betray us into a palpable error, either from want of discernment, or from design, to raise his own importance. This passage of Scripture explained by Mead, only forms a part of his beautiful whole. All the causes are enumerated, and consequently it is no proof that this obtains in old age more frequently than all the others. But Mr. Everard Home, if he had not pilfered this from Dr. Mead, would not have been the first among those that quoted Scripture for his purpose; the Devil has been said always to have done so.

"If we were tempted to put Dr. Mead's paraphrase out of the question, and were left to a barbarous construction, the variations would be curious. Perhaps Mr. Everard Home would define the silver chord to be the spermatic chord, his attention being directed so so partially to those parts; whilst another may define it to be heart, nerve, or tendon. Perhaps, if Mr. Everard Home should hereafter prosecute a closer study of this allegory, and no person is fitter for it, if his zeal should receive the touch of hallowed inspiration, if he could but open a spiritual correspondence with the Sergeant Surgeon of King Solomon, the whole of the mysteries of the passage might be truly explained, for Dr. Mead's might be mere conjecture after all. Who is there so likely to be inspired, and to inspire a Sergeant Surgeon, as Mr. Everard Home? As he has been so successful in his dealings with the Sergeant Surgeon of a present king, he is the likeliest, with the aid of inspiration, to succeed with the Sergeant Surgeon of a past. His is the 'voice of the charmer.'"

Upon the dereliction of caustic, by Sir Everard Home, in diseases of the urethra, the Reviewer is particularly severe; and, upon the motives which have induced to the publication of the "Practical Observations," he writes with a freedom and hardy boldness that must be our excuse for not inserting the passage.

Upon the discovery itself, and upon the methods of treating diseases of the Prostate, we have some satisfaction in quoting more largely, because it will enable us to lay before our readers a short history of the progress of surgical knowledge on this most interesting subject.

"Mr. Everard Home presumes that he has established 'this newly-acquired anatomical fact;' and he tells us, in p. 13, that neither Winslow, Haller, nor Morgagni, knew of it. The truth of the matter is, that Haller denied that the prostate gland was a lobulous gland, after it had been asserted by another like Mr. Everard Home. Haller says, 'glandula aut certè cellulosum compactum corpus dicitur.' Now there is a very wide difference indeed between a person not finding out any fact not already discovered, and giving his assent or dissent to one that is asserted to be discovered. If a person has pointed out a fact, it must be seen; but, if a person points out as a fact, such as what Mr. Everard Home has asserted, as to the prostate gland being lobulous, and the middle lobe a part of it; and if, after that, it, notwithstanding, cannot be seen, it must be confessed to be more properly deemed a fiction than a fact. Therefore, from henceforth, especially as no one anatomist, since nor before, past nor present, has asserted that the prostate gland is lobulose, and that this middle part is a lobe of it under any construction of partial attachment, especially as every day since his avowal of this discovery six years ago in the Philosophical Transactions, a free opportunity has been given for the confirmation or the denial of his discovery, and especially as it is universally since denied that the prostate is lobulose, it cannot be any thing more or less than a fiction.

"Mr. Everard Home has referred us to Morgagni, and, with an air of triumph, he exults that Morgagni had not forestalled him in his newly-discovered anatomical fact. Few writers mention authors to say that their works do not contain any thing to the purpose. But, if if Hunter, Desault, and Morgagni, have not satisfied Mr. Everard Home's curiosity, why did he not extend his researches? we mean with respect to the history of disease of the prostate, and of cases; for, as to the discovery of the middle lobe, we freely confess that to be all his own. But, as he would not look out further for these, we have to inform him that the disease of the prostate gland, and its relief by the catheter, have been well and truly defined for more than two hundred years. Disease of the prostate also can be easily ascertained. There is no disease that can be more readily distinguished, either from stricture in the urethra, or from various attacks of the bladder, ureters, or kidneys. There are wanting but a very few minutes for a surgeon to decide whether the prostate be diseased or not. That which has mostly attracted the attention of surgeons, are the means for relief of patients in diseases of these parts, of the urethra, the prostate, bladder, ureters, or kidnies. And, in his history of the treatment of these diseases by others, Mr. Everard Home has taken especial care to be studiously as close as possible: he has never pointed out, nor referred us to, any others but to Hunter and Desault, and he would have it insinuated that these diseases, which are natural to, and hereditary with, our frame, had gone on without a case, a comment, or a single consideration, but from Hunter or Desault. This I conceive to be, if it were true, a scandal to the profession. The intention of this review will not permit me to set about collecting cases, in order to establish my assertion by many examples, that cases of diseases of prostate and bladder, and their treatment, are very common, and easily found. I shall rather refer the reader, for the sake of brevity, to what a few modern and antient writers have said upon the subject, to shew how perfectly true these diseases have been all along defined and treated. I could have given innumerable cases, if that were necessary; let others be but content to take advantage of my references in aid of their own researches, and they cannot fail of receiving conviction. I shall, therefore, henceforth dismiss the middle lobe, and give a few comparative observations from other authors, as to their knowledge of the prostate in every state, sound as well as diseased, and particularly as to the use of the catheter. Among other cases, for example, I refer to Fothergill's, by Lettsom.

"Mr. Everard Home has been diffuse upon his preferable mode of relieving the bladder in cases of diseased prostate; and yet, to do him justice, although he has changed his practice, and substituted the flexible catheter for the metal one; although he has thus capered and vaunted about the flexible catheters being kept for ten years for their amendment; although we all know this to be one of his flowers of writing; and, although it would not have suited his interest, nor his genius, to have preferred a common and a most useful instrument, such as the elastic gum catheter is, without annexing to it a false quality for his own purpose, yet I will not hesitate to say, that no one but a surgeon could have done what he has done so well. In making my references to modern authors for their comparative merits, I naturally, amongst the best of them, perused the performances of Mr. Brandon Trye and Mr.

Walter

Walter Weldon, published about twenty years ago. Every expression referable to the drawing off the urine had been impressed upon my memory; and, on reading Mr. Everard Home's work, I could turn from his to Mr. Walter Weldon's, and confirm passages in his, which Mr. Everard Home must have referred to. He has gone further than this, and afforded yet a stronger corroboration of my assertion: he has taken a simile from Mr. Walter Weldon's book, but, lest it might be thought too palpable, he has changed the thought from a painter to an engraver, and, as might be expected from him, he has clothed it with coloring and varnish of his own. I will now put him along side, for once, of a modest and ingenious writer.

"Mr. Walter Weldon says, in p. 171, 'the catheter in the hand of a surgeon, like a pencil in the hand of a painter, requires frequent use and much practice to be managed with facility and success.'

Mr. Everard Home says, in p. 89, 'an eminent engraver, on whom I passed a catheter, after unsuccessful attempts had been made by others, told me, that he saw by the light mode of handling the instrument that I was master of it; and that in his own art the graver required a management in the hand which it is difficult to learn, and only to be acquired by practice.'

"This performance of Mr. Weldon's was published in 1792. It was written on a purposed subject of prize dissertation, when he was member of a society in Southwark. It was dedicated to Mr. Thompson Forster. The practice here displayed was the result of the knowledge of the surgeons and pupils of St. Thomas's and Guy's Hospitals. It is a pure and perspicuous production. The extract I am about to give from it applies to his description of a diseased prostate producing a suppression of urine.

"'P. 105. There is, however, a disease of this part, which is frequently the cause of a retention of urine. At what time of life this disease generally first commences, I cannot say; but that state of it which gives rise to a retention of urine, is by much the most frequent in old people, though not peculiar to them: I have seen it in a person below the age of forty. In this state, the prostate gland is six or eight times its natural size. Instead of lessening, it increases the size of that portion of the urethra which is situated within it, rendering it both wider and longer, but at the same time increasing its incurvation.

"'In consequence of its increased size, and of the change it undergoes in form, the sides of it, and its posterior portion, extend into the cavity of the bladder, giving the appearance of two or three tumors, sometimes of equal, sometimes of different, sizes. These, falling together, act as valves to the urethra, preventing the free evacuation of the urine. They are so situated as to fall together spontaneously; the consequence of which is, that they are pressed together with greater force, in proportion as the bladder becomes distended with urine. A person will sometimes bear this for several years, without any very great inconvenience, provided he is careful to void his urine frequently; but, if he neglects it for some time beyond the usual period, he will not be able to void it at all.'

"Without the machinery of the middle lobe, Mr. Weldon could describe the protrusion of an enlarged prostate, pressing on the bladder. He could describe three tumors thrown out from the diseased posterior portion of the prostate forming into one. Mr. Weldon knew what we all are in general apprised of, that the figure of a sound gland is perfectly lost in the appearance of a diseased one. I wish Mr. Everard Home had even taken more slices from this performance of so much genuine utility; the form of the catheter, and the passing it, are much better laid down by Mr. Weldon than by him.

"Modern writers on surgery and anatomy compose and publish on single subjects. I do not forget the generalisation of some, but I consider them merely as compilers. The productions are therefore scattered, and cannot be so readily referred to, as more antient ones can. Ancient writers treat on diseases of parts, at the time when they are describing the situation, the anatomy, and the uses, of them. Their works, therefore, can always be referred to; and long before Morgagni's time, to which Mr. Everard Home confined his researches, enough is to be found upon diseases of the prostate gland. Perhaps Morgagni, who died no later than 1771, said less, because so much had been well said before. Mr. Everard Home's conclusion from Morgagni's silence upon the subject, might be just the contrary of what it should have been; that the subject was not dark with the antients, but that their sight of it was so clear that he had nothing better to offer; and this is not difficult to be proved.

"Bonetus de Abditis Morborum Causis per Cadaverum Dissectionem revelatis, Geneva edition, folio, printed in 1700, p. 632, *de Urinæ Suppressione,* has given columns upon the subject. The following is a statement of one case, out of many more, from an examination after death.

"'Corpore à P. de Marchettis eviscerato, renes cæteræque generationis partes rectè se habuerunt: soli vesicæ orificio appendicula callosa interius adnata, et verius interior ambitus orificii membranosus, in articuli magnitudinem excreverat, quæ antehac mictionem cæteroquin difficilem, sensim producens, affluente post muco pituitoso, sic viam angustavit ut ne catheterem quidem admiserit: vesica erat lotio repleta rite pariter præclusa inferiore via: vesica quinetiam, cum intestinis flatibus turgens, ab extensione adeò extenuata ut contactu fuerit disrupta: inflammatio nulla initio, nec febris notata, quam progressu morbi deprehendimus.'

"One hundred years before Bonetus, and long, very long, before Wiseman, the physicians, who at that time were many of them the most eminent anatomists of their day, have faithfully and amply recorded histories of the symptoms and examinations of the state of the parts in diseases of the prostate gland, before and after death."

To these authorities the Reviewer has annexed the detail of a case from the Bibliotheca Anatomica, (*fol. Genev.* 1685,) in which it was inserted from the works of Dr. Theodore Mayerne. This case has been on record 200 years, is of such importance from the facts it contains, and is of such interest from the celebrated person who was the subject of it, that, notwithstanding we have given more space than usual to this part of our Journal, we cannot suppose our our readers will be dissatisfied with its insertion, in the translation of the Reviewer.

"The illustrious Isaac Casaubon was naturally of a spare and thin habit, and of a bilious temperament approaching to melancholic. A mind of divine energy and vigor was imprisoned a guest in a frame of very inadequate dimensions, and, entirely devoted to letters, bestowed but little attention on the body that sustained it. His health, therefore, not merely was impaired, but almost destroyed, by literary abstraction. The earlier years of his life were not embittered by any very violent disease; and such was the regularity of his habits, that he then felt little of the inconveniences of a sedentary life, his urine sometimes deposited a sabulous matter, with occasional heat in the act of voiding it, but not in a sufficient degree to dwell upon or to require the frequent attention of his physicians; he changed his abode and climate often, so that his mode of diet also was various at different periods; often did he pass very many hours in his studies forgetful of food, and whole nights in polemic writing. At length his spirits, strength, and corporeal functions, became deranged, and nature impeded and fatigued in her operations. Twelve months before his death he began to complain of lancinating pains when he passed his urine, and the heat also which he had complained of returned; first at longer intervals, but afterwards interrupting his sleep by the necessity of urining. These symptoms were attributed to gravel, but nothing could abstract his attention from his studies, till at length the difficulty of urining increased, and he could not effect it without effort, and curving the body downwards. So great was the irritation, that he who seldom emptied his bladder during the night, was in a few months compelled to rise repeatedly, and at last innumerable times, for that purpose. Pains in the groin, a sensation of weight at the anus, and inclination to stool at the time of urining, lancinating pains through the urethra, but particularly in the glans, were also complained of; sometimes a violent heat was felt in the back, so great that he sought relief from taking off his clothes. When he used exercise, either of walking or on horseback, he voided pale mucous urine, and small calculi, some of them as large as a grain of wheat, which led his physicians to suppose that his chief complaint was stone in the bladder. To ascertain this, when an attempt was made to introduce the catheter, it was obstructed by very evident marks of hypersarcosis in the canal. Added to this numerous list of painful symptoms, which was enough to weaken the strongest frame, six months before he died there appeared in the left hypochondrium, between the bladder and the os ilium, an inflamed tumor, the size of a walnut, increasing to the size of the fist, of an obtuse form, and which appeared on feeling it to contain fluid. From the free action of the abdominal muscles, in coughing and respiration, it seemed that the fluid was under the abdominal muscles; not however in the cavity of the abdomen, but as if contained in a cyst. If any degree of pressure were made on the tumor, the bladder became painful, with efforts to make water, so that it seemed to be situated under the peritoneum. It sometimes subsided, but in a few days returned to its former size. In this miserable state he languished for several months, and his approaching death was accelerated, when, after riding several miles in a carriage for exercise on a fine day, on his return home his painful micturition and strangury increased, and a large quantity of tenacious bloody mucus, like pus, was mixed with his urine. The tenesmus increased, small calculi were voided; in short, all his complaints became worse, and accompanied with fever, and every means were used in vain for his relief. The mucous urine flowed involuntarily with great heat; and what should be remarked, this mucous deposit was sometimes absent for some days and returned again. With these dreadful sufferings he struggled for about three weeks, when his spirit, impatient of its prison, was summoned by the Supreme Being, to return to that heaven from whence it was derived. He died July 1614, aged 57.

"On opening his body, the following appearances presented themselves to our careful examination. A catheter, introduced into the urethra, could by no force or art be carried into the bladder; but, on opening the perineum, a fleshy excrescence manifestly appeared, occupying the passage, preventing the entrance from without, but allowing an instrument to pass with difficulty from within. The prostate gland was four times larger than its natural size. The bladder was thickened, contracted, and its internal surface furrowed as if it had been done with instruments, and in the sulci there were many calculi. The neck of the bladder, which naturally terminates smoothly, and without any protuberance in the urethra, was tumid within like the rump of a fowl, or the interior of the os uteri, so that the finger might be passed into a sulcus or groove surrounding it.

"The bladder was perforated on its left side by a hole which would admit four fingers into a sac or process from the bladder, or like another bladder, which, when full, would contain six times as much as the bladder itself. This sac was composed of three coats continued from the bladder and some tendinous fibres; it had also nerves, veins, and arteries.

"It occupied the whole hypogastric region on the left side, even to the os ilium, like a bladder with its peritoneum. Its figure was unequal, and the lower part depended beyond the aperture, and the upper (a larger part) was annexed to the peritoneum. On the base of the sac, below the communication, there was a quantity of that putrid mucus or matter, which was passed with such pain and difficulty from the patient when alive. The bladder was neither ulcerated, nor much inflamed. The internal membrane of the sac was eroded, livid, and gangrenous in many places, which seemed to have been the cause of his death. The ureters were large, and distended with urine. The left one passed between the coats of the sac, but they both terminated in the bladder itself a little above the neck. The right kidney was purulent, the left sound. The other parts were in a sound state, except the lungs, which had suffered from a long catarrhal affection, which almost deprived him of his voice."

